# Therapies to Restore Consciousness in Patients with Severe Brain Injuries: A Gap Analysis and Future Directions

**DOI:** 10.1007/s12028-021-01227-y

**Published:** 2021-07-08

**Authors:** Brian L. Edlow, Leandro R. D. Sanz, Len Polizzotto, Nader Pouratian, John D. Rolston, Samuel B. Snider, Aurore Thibaut, Robert D. Stevens, Olivia Gosseries, the Curing Coma Campaign and its contributing members, Yama Akbari, Thomas P. Bleck, Michael N. Diringer, Brandon Foreman, Jed A. Hartings, Raimund Helbok, J. Claude Hemphill, Geoffrey S. F. Ling, Stephan A. Mayer, Molly McNett, Martin M. Monti, DaiWai M. Olson, Adrian M. Owen, Soojin Park, J. Javier Provencio, Louis Puybasset, Paul Vespa, Amy Wagner, John Whyte, Wendy Ziai

**Affiliations:** 1 Center for Neurotechnology and Neurorecovery, Department of Neurology, Massachusetts General Hospital and Harvard Medical School, 175 Cambridge Street – Suite 300, Boston, MA 02114 USA; 2 Athinoula A. Martinos Center for Biomedical Imaging, Massachusetts General Hospital and Harvard Medical School, Charlestown, MA USA; 3 Coma Science Group, GIGA-Consciousness, GIGA, University of Liege, Liege, Belgium; 4 Centre du Cerveau, University Hospital of Liege, Liege, Belgium; 5 Department of Biomedical Engineering, Worcester Polytechnic Institute, Worcester, MA USA; 6 Department of Neurosurgery, David Geffen School of Medicine, University of California, Los Angeles, Los Angeles, CA USA; 7 Departments of Neurosurgery and Biomedical Engineering, University of Utah, Salt Lake City, UT USA; 8 Department of Neurology, Brigham and Women’s Hospital and Harvard Medical School, Boston, MA USA; 9 Departments of Anesthesiology and Critical Care Medicine, Radiology, and Neurology and Neurosurgery, School of Medicine, Johns Hopkins University, Baltimore, MD USA

**Keywords:** Coma, Consciousness, Disorders of consciousness, Gap analysis, Precision medicine

## Abstract

**Background/Objective:**

For patients with disorders of consciousness (DoC) and their families, the search for new therapies has been a source of hope and frustration. Almost all clinical trials in patients with DoC have been limited by small sample sizes, lack of placebo groups, and use of heterogeneous outcome measures. As a result, few therapies have strong evidence to support their use; amantadine is the only therapy recommended by current clinical guidelines, specifically for patients with DoC caused by severe traumatic brain injury. To foster and advance development of consciousness-promoting therapies for patients with DoC, the Curing Coma Campaign convened a Coma Science Work Group to perform a gap analysis.

**Methods:**

We consider five classes of therapies: (1) pharmacologic; (2) electromagnetic; (3) mechanical; (4) sensory; and (5) regenerative. For each class of therapy, we summarize the state of the science, identify gaps in knowledge, and suggest future directions for therapy development.

**Results:**

Knowledge gaps in all five therapeutic classes can be attributed to the lack of: (1) a unifying conceptual framework for evaluating therapeutic mechanisms of action; (2) large-scale randomized controlled trials; and (3) pharmacodynamic biomarkers that measure subclinical therapeutic effects in early-phase trials. To address these gaps, we propose a precision medicine approach in which clinical trials selectively enroll patients based upon their physiological receptivity to targeted therapies, and therapeutic effects are measured by complementary behavioral, neuroimaging, and electrophysiologic endpoints.

**Conclusions:**

This personalized approach can be realized through rigorous clinical trial design and international collaboration, both of which will be essential for advancing the development of new therapies and ultimately improving the lives of patients with DoC.

**Supplementary Information:**

The online version contains supplementary material available at 10.1007/s12028-021-01227-y.

## Introduction

Treatments for patients with disorders of consciousness (DoC) are currently limited. The cornerstone of therapy is early intensive neurorehabilitation combining physical, occupational, speech/language, and neuropsychological therapy, which appear to improve long-term functional recovery [1–4]. Pharmacologic stimulant therapies are also used throughout the rehabilitation process to promote recovery of consciousness [5]. However, of the few rehabilitative or pharmacologic therapies that have reached late-phase clinical trials, only amantadine has evidence from a multicenter, double-blind randomized controlled trial to support its efficacy in accelerating recovery in patients with posttraumatic DoC [6–8].

Network-based insights into mechanisms of consciousness [9] now raise hope for developing new consciousness-promoting therapies for patients with DoC [5]. A fundamental goal is to modulate the neural networks underlying arousal and awareness, the two components of consciousness [10]. Central to this effort has been the development of network-based conceptual models of consciousness [11, 12] as well as methodologic advances in neuroimaging [13], electrophysiology [14], and neuromodulation [15, 16]. These conceptual and methodologic advances now make it possible to test precision therapies [17, 18] that modulate brain activity at a range of scales [19–22].

Yet measuring the effects of therapies remains a challenge. Even with advances in the bedside assessment of patients with DoC [23, 24], consciousness may evade detection by behavioral examinations, and thus therapeutic effects may go unnoticed. The recognition that up to 15–20% of patients who appear unresponsive may be covertly conscious [25–29] (i.e., cognitive motor dissociation [30]) has led to a reappraisal of behavioral outcome measures in clinical trials and a search for new electrophysiologic and imaging biomarkers of therapeutic efficacy [17, 18]. Furthermore, the optimal time window for evaluating efficacy has not been defined because some treatments produce an immediate, transient effect on a patient’s level of consciousness, whereas others may cause a delayed, long-term change in a patient’s course of recovery.

In this white paper, we report the results of a gap analysis performed by the Coma Science Work Group of the Curing Coma Campaign [31], in which we examine therapies that aim to promote recovery of consciousness in patients with DoC. We identify gaps in knowledge that have impeded the development of effective therapies, and we propose strategies for filling these gaps in future clinical trials. We make suggestions for the development and rigorous assessment of new therapies based on emerging insights into mechanisms of consciousness and its disorders.

## Work Group Meetings and Literature Review

The Curing Coma Campaign convened a Coma Science Work Group that included nine clinicians and neuroscientists with expertise in DoC. The work group represented six international academic medical centers and the fields of neurology, neurosurgery, physical medicine and rehabilitation, neuropsychology, and neuroscience. The work group met online biweekly and performed a gap analysis over a 6-month period from June to December 2020. During this period, we reviewed the literature on therapies for DoC using reference libraries from recent systematic reviews [5, 10, 32] as well as our own reference libraries. We focused on therapies that directly modulate brain networks involved in human consciousness. We therefore did not consider brain–computer interfaces that translate neural activity into self-expression [14, 33], nor did we cover treatments that ameliorate specific symptoms or neurological deficits associated with DoC, such as spasticity [34], pain [35], dysautonomia [36], nonconvulsive seizures [37], pituitary failure [38], or hydrocephalus [39]. Although the successful treatment of such symptoms can facilitate self-expression and reduce confounding of behavioral assessments [1], these treatments were beyond the scope of the present gap analysis.

**Fig. 1:**
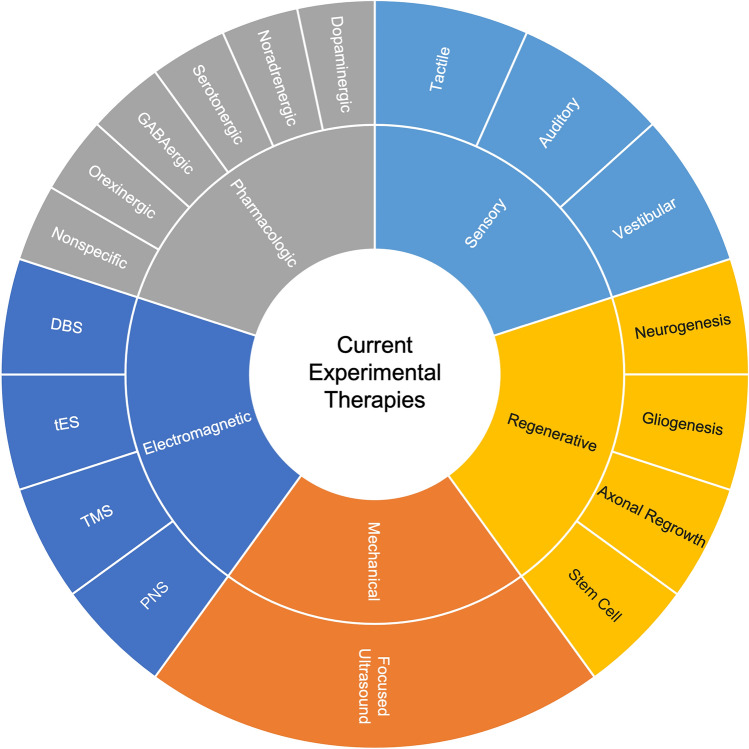
Current experimental therapies for patients with disorders of consciousness. The therapies are color-coded according to the five classes we identified in the gap analysis: (1) pharmacologic, (2) electromagnetic, (3) mechanical, (4) sensory, and (5) regenerative. *DBS* deep brain stimulation, *PNS* peripheral nerve stimulation, *tES* transcranial electrical stimulation, *TMS* transcranial magnetic stimulation

We categorized current experimental therapies into five types: (1) pharmacologic, (2) electromagnetic, (3) mechanical, (4) sensory, and (5) regenerative (Fig. 1). These therapeutic classes act via distinct mechanisms, with a diverse set of stimulation targets (Table 1). We summarized the state of the science for each therapy then analyzed current gaps in knowledge (Table 2) and proposed future experimental directions (Table 3). All recommendations by our work group were based on consensus agreement. We focused on how the design of future clinical trials can be optimized for patients with DoC, recognizing that recent innovations in clinical trial design, such as adaptive designs [40] and patient-centered outcomes [41, 42], are likely to also influence future trials of consciousness-promoting therapies.

**Table 1:** Putative network targets for experimental therapies aimed at promoting recovery of consciousness

Target network	Network nodes	Pharmacologic	Electromagnetic	Mechanical	Sensory	Regenerative
Ascending arousal network	mRt, VTA, LC, PTg, PnO, PBC, LDTg, DR, MnR, PAG, IL, Ret, TMN, LHA, SUM, NBM, DBB	DA, NE, 5HT, ACh, Glu, GABA, Ox, nonspecific	DBS, PNS	LIFUP	Vestibular, tactile, auditory	Stem cells, neurogenesis, gliogenesis, axonal regrowth
Default mode network	PCC, Pr, vMPFC, dMPFC, IPL, HF, LTC, Th		TMS, tES	LIFUP	–	
Salience network (ventral attention network)	dACC, FI, AI, SLEA, PAG, TP, SN, VTA, Hy, Put, dmTh, antTh		–	–	Auditory, tactile, vestibular	
Dorsal attention network	FEF, IPS, SPL, aMT		–	–	–	
Executive control network (frontoparietal network)	dLPFC, dMPFC, vLPFC, LP, dCN		TMS, tES	–	–	
Thalamocortical network	IL, cerebral cortex		DBS	LIFUP	–	
Limbic network	OF, TP		–	–	Auditory	
Somatomotor network	S1, M1, SMA, PMC		TMS, tES, PNS	–	Tactile, vestibular	
Visual network	V1, V2, V3, V4		–	–	–	
Auditory network	STG, IFG		–	–	Auditory	

Canonical neural networks that have been characterized in the human brain are listed in the first column. Network nodes and neuroanatomic abbreviations are listed in the second column, based upon recent network-based studies [235–240]. The five types of therapeutic modalities characterized in this gap analysis are listed in subsequent columns, and the putative network targets of each therapy are listed in the individual cells of the table. Of note, there are ongoing debates about the incorporation of specific nodes in certain networks (e.g., the inclusion of the thalamus in the DMN)

*5HT* 5-hydroxytryptamine (serotonin), *ACh* acetylcholine, *AI* anterior insula, *aMT* anterior middle temporal area complex, *antTh* anterior thalamus, *DA* dopamine, *dACC* dorsal anterior cingulate cortex, *DBB* diagonal band of Broca, *DBS* deep brain stimulation, *dCN* dorsal caudate nucleus, *dLPFC* dorsolateral prefrontal cortex, *DMN* default mode network, *dMPFC* dorsomedial prefrontal cortex, *dmTh* dorsomedial thalamus, *DR* dorsal raphe, *FEF* frontal eye fields, *FI* frontoinsular cortex, *GABA* γ-aminobutyric acid, *Glu* glutamate, *Hy* hypothalamus, *HF* hippocampal formation, *IFG* inferior frontal gyrus, *IL* intralaminar nuclei of thalamus, *IPL* inferior parietal lobule, *IPS* intraparietal sulcus, *LC* locus coeruleus, *LDTg* laterodorsal tegmental nucleus, *LHA* lateral hypothalamic area, *LIFUP* low-intensity focused ultrasound pulsation, *LP* lateral parietal cortex, *LTC* lateral temporal cortex, *M1* primary motor cortex, *MnR* median raphe, *MNS* median nerve stimulation, *mRt* midbrain reticular formation, *NBM* nucleus basalis of Meynert, *NE* norepinephrine, *OF* orbitofrontal cortex, *Ox* orexin, *PAG* periaqueductal gray, *PBC* parabrachial complex, *PCC* posterior cingulate cortex, *PMC* premotor cortex, *PnO* pontis oralis (i.e., pontine reticular formation), *PNS* peripheral nerve stimulation, *Pr* precuneus, *PTg* pedunculopontine tegmental nucleus, *Put* putamen, *Ret* reticular nucleus of the thalamus, *S1* primary somatosensory cortex, *SLEA* sublenticular extended amygdala, *SMA* supplementary motor area, *SN* substantia nigra, *SPL* superior parietal lobule, *STG* superior temporal gyrus, *SUM* supramammillary nucleus of the hypothalamus, *tES* transcranial electrical stimulation, *Th* thalamus, *TMN* tuberomammillary nucleus of the hypothalamus, *TMS* transcranial magnetic stimulation, *TP* temporal pole, *vLPFC* ventrolateral prefrontal cortex, *vMPFC* ventromedial prefrontal cortex, *V1, V2, V3, V4* primary and association visual cortices, *VTA* ventral tegmental area

**Table 2:** Overview of experimental therapies for DoC

Class of therapy	Pharmacologic	Electromagnetic	Mechanical	Sensory	Regenerative
Current modalities	DA, NE, 5HT, ACh, Glu, GABA, Ox, nonspecific	DBS, tES, TMS, PNS	LIFUP	Tactile, auditory, vestibular	Stem cells, neurogenesis, gliogenesis, axonal regrowth
Highest level of evidence	RCT (amantadine) [6]	RCT (tDCS, TMS) [126, 133]	Case report/series [190, 191]	RCT (auditory) [194, 198]	Phase 1 clinical trials (stem cells) [213, 214]
Treatment efficacy	Faster rate of recovery during a 4-week treatment period (amantadine)	New signs of consciousness in 30–50% of patients in MCS (frontal tDCS), behavioral improvement in open-label studies, no RCT evidence of efficacy yet (TMS)	Behavioral improvement in 1 acute patient and 2 of 3 chronic patients	Behavioral improvement, increased fMRI activation, higher interactive autonomic activity (auditory)	Possibly faster rates of clinical improvement (stem cells)
Safety	Mild common and rare severe adverse events	DBS, invasive VNS: rare severe adverse events; tES: mild adverse events; TMS: mild adverse events and rare seizures	Physical discomfort, modulation of unintended targets	Sensory: no reported adverse effects; vestibular: mild adverse effects	Unknown safety profile, potential infusion site reactions and malignancies
Limitations	Delayed action, drug tolerance, transient effects	DBS, invasive VNS: cost and access; tES, TMS: moderate and transient effects	Early development for DoC	Tactile and auditory: uncertain efficacy; Vestibular: early development for DoC	Early development for DoC
Ongoing clinical trials^a^	4	10	1	5	0
Gaps in knowledge	Linking functional networks to individual neurotransmitters, measuring neurotransmitter imbalances, identifying likely responders to therapy	Mechanism of action on neural networks, excitability and plasticity, optimal stimulation parameters and sites, contact localization, benefits of concurrent medications	Optimal anatomical targets, stimulation paradigms, benefits of adjuncts, system design for clinical use	Unknown mechanisms of action, limited knowledge on vestibular cortical representation	Integration of stem cells into damaged networks

*5HT* 5-hydroxytryptamine (serotonin), *ACh* acetylcholine, *DA* dopamine, *DBS* deep brain stimulation, *DoC* disorders of consciousness, *fMRI* functional magnetic resonance imaging, *GABA* γ-aminobutyric acid, *Glu* glutamate, *LIFUP* low-intensity focused ultrasound pulsation, *MCS* minimally conscious state, *NE* norepinephrine, *Ox* orexin, *PNS* peripheral nerve stimulation, *RCT* randomized controlled trial, *tDCS* transcranial direct current stimulation, *tES* transcranial electrical stimulation, *TMS* transcranial magnetic stimulation, *VNS* vagus nerve stimulation

^a^We performed a search on ClinicalTrials.gov on January 15, 2021, for interventional clinical trials on the condition “disorder of consciousness,” with a status of “recruiting,” “active, not recruiting,” or “enrolling by invitation.” This search returned 69 results, of which 20 were included in one of five classes of therapeutic modalities and 49 were excluded (39 with a non-DoC population, 6 non-interventional, and 4 without direct action on consciousness). Please see Supplementary Table 2 for additional details regarding the clinical trials identified by this search

**Table 3:** Future goals for the development of therapies to promote recovery of consciousness

Goal	Action items
Goal 1: develop a unifying conceptual framework for therapeutic mechanisms of action	Create network-based models of arousal and awareness, the two components of consciousness Validate new electrophysiologic and imaging tools to map brain network connectivity
Goal 2: optimize the design of clinical trials	Perform double-blinded, placebo-controlled, randomized studies with large sample sizes Implement advanced clinical trial designs, such as adaptive designs Develop patient-centered outcome measures in partnership with families and caregivers Establish an operational framework for enrolling patients with CMD (i.e., covert consciousness) and for measuring CMD as an outcome
Goal 3: select patients for clinical trials on the basis of a precision medicine approach	Tailor therapies to individual genomic, proteomic, and metabolomic profiles Enrich patient selection for clinical trials by enrolling patients whose brain network connectivity suggests a physiologic receptivity to therapeutic intervention Define patient-specific endotypes in the inclusion and exclusion criteria of clinical trials
Goal 4: develop pharmacodynamic biomarkers of therapeutic responses	Measure surrogate biomarkers of a subclinical brain response in early-phase trials Characterize intrasubject and intersubject variance in biomarker responses
Goal 5: determine the optimal timing and dosing of therapeutic interventions	Characterize the temporal dynamics of brain network receptivity to neuromodulation during the acute, subacute, and chronic stages of recovery from brain injury Determine if a patient’s endotype influences the therapeutic window or duration of action Measure neurotransmitter function within specific brain networks that are therapeutic targets Optimize the neuroanatomic precision of targeted invasive and noninvasive therapies Identify the optimal stimulation targets within widely distributed neural networks
Goal 6: develop novel combination therapies	Test the efficacy of concurrent therapies from different modalities (e.g., pharmacologic and electrophysiologic) Test the efficacy of concurrent therapies from the same modality (e.g., top-down and bottom-up electrophysiologic stimulation)
Goal 7: establish an international clinical trials network	Create global collaborations to support large-scale phase 3 clinical trials

Goals are listed according to the order that they appear in the text

*CMD* cognitive motor dissociation

## Pharmacologic Therapies

### Pharmacologic Agents: State of the Science

Several classes of pharmacologic agents have been used to promote recovery of consciousness in patients with DoC [5, 43]. Treatment selection has been guided by the observation that multiple neurotransmitter systems contribute to human consciousness [44, 45] and are disrupted by brain injury [46, 47]. Animal and human studies have revealed abnormal levels of glutamate, dopamine, acetylcholine, γ-aminobutyric acid (GABA), and orexin after brain injury [48–53], although the precise mechanistic role of each neurotransmitter system in consciousness is not fully understood. Overall, there are limited data about pharmacologic interventions for patients with DoC, with notable exceptions below.

Dopaminergic drugs have received particular attention because dopamine is a stimulatory neurotransmitter that is widely expressed in the human brain, including in the anterior forebrain mesocircuit [54–56], a network of cortico-subcortical feedback loops that appears to be essential in the alteration and recovery of consciousness [57]. Brain injury causes widespread deafferentation and neuronal death within the anterior forebrain mesocircuit, which causes dysfunction within striato-thalamocortical feedback loops, as demonstrated by growing neuroimaging evidence [57–59]. Dopamine appears to regulate the activity of the mesocircuit and promote clinical recovery because it facilitates the output of striatal neurons to the globus pallidus and directly modulates the mesiofrontal cortex, leading to restored forebrain activity [56]. Accordingly, behavioral and neuroimaging responses have been observed following the administration of dopaminergic agents to patients with prolonged DoC [55]. Levodopa [60, 61], bromocriptine [62], apomorphine [63, 64], and methylphenidate [65] have been investigated in small studies that preclude definitive conclusions regarding efficacy. However, amantadine has been tested in a placebo-controlled, randomized, double-blind trial in 184 patients 1–4 months after severe traumatic brain injury [6]. This trial revealed a significantly higher rate of behavioral recovery among the amantadine group during treatment, which declined below the rate of the placebo group during the washout phase. Amantadine is currently the only therapy recommended in the 2018 DoC guideline endorsed by the American Academy of Neurology, American Congress of Rehabilitation Medicine, and National Institute for Independent Living and Rehabilitation Research [7, 8].

Paradoxically, the sedative zolpidem has also demonstrated stimulating effects in a small subset of patients with DoC [66, 67]. Its modulation of GABA_A_ receptors in the globus pallidus interna is thought to underlie transient behavioral improvements through release of inhibition on the mesocircuit [56, 68, 69]. One double-blind, placebo-controlled crossover study in 84 patients in a vegetative state/unresponsive wakefulness syndrome (VS/UWS) or a minimally conscious state (MCS) identified 5% of patients as “definite responders” [70], whereas another prospective open-label trial in 60 patients with DoC showed behavioral improvements in 20% of patients, without a change in level of consciousness [71]. Zolpidem responses have been associated with regional increased metabolism on fluorodeoxyglucose positron emission tomography [72], an increased blood–oxygen level-dependent signal on functional MRI (fMRI) [73], reduced burst suppression on electroencephalography (EEG) [74], and restoration of thalamocortical signaling on dynamic EEG analyses [68, 69, 75].

Other types of pharmacologic drugs, such as baclofen (GABA_B_) [76–78], midazolam (GABA_A_) [79], amitriptyline [80], desipramine, protriptyline [81] (norepinephrine and serotonin), and modafinil [82] (norepinephrine, dopamine, and orexin), have also shown variable benefit in small-sample studies. It is unknown whether the use of multiple stimulants in combination provides therapeutic benefit over use of a single stimulant [83]. Additionally, new types of drugs are emerging as potential candidates to promote recovery of consciousness. For instance, psilocybin, which binds to serotonin receptors, is thought to increase the complexity of electrophysiologic brain measures in healthy controls [84] and could thus promote behavioral responsiveness in patients with DoC whose EEG demonstrates decreased brain complexity [85, 86].

Adverse effects are a concern in this vulnerable population, and pharmacotherapies may be associated with mild reactions (e.g., agitation, hypertension, tachycardia, rash, sleep disturbances, vomiting) or, rarely, severe side effects (e.g., seizure, arrhythmia) [87, 88]. Importantly, adverse reactions to pharmacotherapy in patients with DoC can vary in type and incidence from those observed in the population in which the drug’s safety was initially tested [89]. Neurostimulant efficacy may also be limited by delayed effect, short duration of action, low central nervous system (CNS) penetration, and tolerance, requiring larger or more frequent doses and narrowing the therapeutic window.

### Gaps in Knowledge

Although pharmacologic agents represent a promising therapeutic approach for patients with DoC, two fundamental limitations have hampered translation. First, we lack a conceptual framework to link the action of individual neurotransmitters to the function of distributed brain networks underlying arousal and awareness. Recovery from different endotypes of DoC may be dependent on neurotransmitter-specific pathways, suggesting a therapeutic opportunity if neurotransmitter activity within these pathways can be measured [53, 90]. Second, we lack a standardized approach to identifying neurotransmitter imbalances amenable to therapeutic modulation. Longitudinal sampling of neurophysiologic and biochemical biomarkers is needed to guide the timing of therapy initiation because excessive neurotransmission and neuronal hyperexcitability during the acute period may transition to a state of depleted neurotransmission and hypoexcitability during the subacute-to-chronic period.

Additionally, the effect size of pharmacologic therapies may be underestimated in clinical trials because only a subset of patients respond, and we are currently unable to identify likely responders at the time of clinical trial enrollment. Incomplete knowledge about the optimal dose, duration, dosing frequency, and formulation of pharmacologic agents may also contribute to the underestimation of their efficacy. Ethical considerations pertaining to enrollment of placebo groups [91, 92] and complex approval procedures for novel molecules have further disincentivized large-scale clinical trials. With a relatively small target patient population [93], the market for research and development of new or repurposed therapies to cure coma is not currently a priority for large pharmaceutical companies.

### Proposal for Future Therapies

Demonstrating the efficacy of new or repurposed pharmacologic agents will require methods for selective enrollment of patients based on their physiological and genetic receptivity to candidate therapies [17, 18, 94, 95]. In addition to improving clinical trial design via selective enrollment, we propose three complementary goals for developing pharmacologic therapies for patients with DoC: (1) combination therapies that provide synergistic effects via concurrent modulation of multiple neurotransmitter systems, (2) new pharmacologic agents (e.g., psychedelic drugs [85], antinarcolepsy drugs, and orexin agonists [96]), and (3) testing of drugs in new settings (e.g., in the intensive care unit or at home). The realization of the first two goals will require a better understanding of how neurotransmitter systems modulate functional brain networks underlying consciousness. Indeed, the development of novel or combination therapies will depend on the activation of functional brain networks by targeting specific neurotransmitters and their receptors. On the other hand, the third goal will require new health care frameworks to test the efficacy of pharmacologic agents in a wider array of settings (e.g., early interventions and long-duration treatments), recognizing that different treatments may be indicated at different stages of recovery.

## Electromagnetic Therapies

### Direct Central Nervous System Stimulation: State of the Science

Direct electrical stimulation of the human CNS began with the nineteenth century investigations of Krause, Horsley, and others [97, 98] and has evolved into advanced techniques, such as deep brain stimulation (DBS) [99], which is now in routine clinical use for a range of conditions. Contemporary CNS stimulation is conducted by using a variety of multicontact electrode arrays capable of generating complex and rapidly alternating voltage fields. Adjustment of different stimulus parameters can produce a spectrum of effects on the underlying neural elements, ranging from activation to depolarization blockade, with network-wide physiological changes. Furthermore, chronic stimulation influences neurotransmitter and growth factor synthesis in ways that are currently under investigation [100–103].

With the intention of improving arousal and awareness, direct CNS stimulation has been applied to a variety of targets in patients with prolonged DoC, including the cervical spine [104], midbrain reticular formation [105, 106], the pallidum [107], nucleus accumbens [108], and the central thalamus [109–111]. These studies enrolled patients with DoC of varying severity resulting from heterogeneous injuries at different postinjury time points and used different stimulation paradigms and treatment durations. In uncontrolled case series of stimulation of the central thalamic nuclei [112] and midbrain reticular formation [105], immediate behavioral arousal responses have been reported (e.g., eye opening, vocalization), along with changes to cerebral blood flow and metabolic rate [105].

Of these targets, DBS of the central thalamic nuclei is one of the most extensively studied, with reports ranging from single patients to larger case series [110–115]. Most are uncontrolled experiments, with the notable exception of a single, rigorously conducted double-blind crossover study of a single subject [110]. In open-label case series, longer-term clinical improvements have been observed after DBS in patients with DoC [112, 114, 116], but these results may have been influenced by biases associated with uncontrolled, unblinded studies.

DBS has been used for decades for other indications, with a well-established safety profile and rare complications. However, because DBS and other forms of direct CNS stimulation involve direct access to the CNS with chronically implanted devices, serious complications can occur, including hemorrhage, seizures, infections requiring system removal, and side effects from unintended stimulation of nearby tissue [117–119].

### Transcranial Electrical Stimulation: State of the Science

Transcranial electrical stimulation (tES) uses weak electrical current (1–2 mA), applied transcranially, to modulate cortical excitability via a top-down process [120]. tES comprises transcranial direct current stimulation (tDCS) (direct, constant current), transcranial alternating current stimulation (tACS) (alternating sinusoidal current at a specific frequency), and transcranial random noise stimulation (sinusoidal current with random amplitude and frequency) [121]. Different types of current have different mechanisms of action, but generally tES techniques are hypothesized to alter the neuronal membrane potential and induce long-term potentiation-like plasticity [120]. tDCS is thought to increase focal cortical excitability under the stimulating electrodes, whereas tACS is thought to entrain neural oscillation to a specific frequency [122, 123].

To date, most clinical trials have studied the ability of tES to ameliorate symptoms or improve function in patients with poststroke motor and language deficits, psychiatric disorders, or chronic pain [124]. Most studies of tDCS in patients with DoC targeted the dorsolateral prefrontal cortex [5]. Randomized controlled trials have reported that 30–50% of patients in MCS, but only a small percentage of patients in VS/UWS, demonstrate new signs of consciousness following prefrontal stimulation [125–129]. Other stimulation sites, including the motor cortex and posterior parietal region, yielded smaller effect sizes compared with prefrontal stimulation [5]. Other paradigms, including tACS and transcranial random noise stimulation, applied to small samples of patients with DoC have been inconclusive [130].

tES is considered to be a safe technique. Adverse effects reported in studies on healthy volunteers include paresthesia, itching, skin erythema, and headache, which all rapidly resolved when stimulation ended. However, some precautions need to be taken in patients with DoC, especially those with a craniectomy or a shunt. The main limitation of tES is currently its moderate and transient clinical effects.

### Transcranial Magnetic Stimulation: State of the Science

Transcranial magnetic stimulation (TMS) consists of an oscillating current passed through a metal coil, which creates a fluctuating magnetic field at the surface of the skull, inducing an electric current in a volume of brain tissue [131]. Like other means of electrically stimulating the CNS, a wide range of stimulation parameters can be adjusted, with some patterns modeled after neural oscillations, such as theta burst stimulation [132]. TMS has been applied over multiple cortical regions, including prefrontal, parietal, motor, and occipital cortices. Evidence of repetitive TMS (rTMS) efficacy has been demonstrated for the following disorders: neuropathic pain, depression, stroke, fibromyalgia, Parkinson disease, multiple sclerosis, and posttraumatic stress disorder [124]. For patients with DoC, a few randomized controlled trials using 20-Hz stimulation over the motor cortex have been conducted, without significant evidence of neurobehavioral improvements [133–135]. Other stimulation sites, including the prefrontal cortex and angular gyrus, have not yet been tested with control groups [136–140]. TMS can also be used in conjunction with EEG as a diagnostic tool to measure brain complexity [86], an approach that holds potential as a neurophysiologic biomarker of treatment effect in patients with DoC [141–143].

The most common adverse effects of rTMS are transient headaches, local discomfort in the targeted area, dizziness, and, very rarely, seizure [144]. It is important to screen for potential (subclinical) seizures in patients with DoC prior to rTMS treatment [145]. As with tES, the main limitations are the moderate and transient behavioral effects.

### Peripheral Nerve Stimulation: State of the Science

Two approaches aimed at stimulating peripheral nerves have been tested to promote recovery in patients with DoC: median nerve stimulation (MNS) and vagus nerve stimulation (VNS). Through multiple synaptic connections, stimulation of primary sensory neurons can induce neuroplasticity within somatosensory networks, modulating network responsiveness [146–148]. Pilot studies of MNS applied to patients with acute brain injury showed that MNS improved the level of consciousness and long-term outcomes [149–151]. A large (*N* = 437) open-label study reproduced these preliminary findings in patients with severe traumatic brain injury, showing better recovery at 6 months in the group that received 2 weeks of MNS compared with the control group [152].

VNS is hypothesized to stimulate brainstem, thalamic, and cortical activity in a bottom-up manner. Invasive VNS, mostly used to treat refractory epilepsy [153], was recently shown to induce recovery of consciousness in a patient in a prolonged VS/UWS [154]. Noninvasive VNS, applied transcutaneously to the auricular branch of the vagus nerve, has also been reported to result in behavioral improvement and increased default mode network connectivity [155]. Subsequently, other uncontrolled case series reported heterogeneous and less clinically apparent treatment effects [156, 157]. However, randomized controlled trials are still lacking, both for MNS and VNS, to determine the efficacy of peripheral nerve stimulation on recovery of consciousness.

As with all noninvasive brain stimulation techniques, MNS and noninvasive VNS are typically well tolerated. Reported side effects are minor. On the other hand, invasive VNS is associated with a risk of adverse events related to surgical implantation (e.g., bleeding and infection). Cost and access to this invasive procedure may also limit its use.

### Gaps in Knowledge

How electromagnetic stimulation precisely affects neural networks is unclear [102] and remains an area of active research [158–164]. Furthermore, the mechanisms by which stimulation modulates the function of distributed networks underlying consciousness are incompletely understood. Adding to these challenges, the parameter space of electromagnetic stimulation is vast [165, 166]. Modern stimulation systems can modulate stimulation amplitude, frequency, and pulse width [167] combined into a variety of stimulus trains and pulse waveform shapes [168] and implemented via current or voltage control [169]. Perhaps most critically, in invasive stimulation techniques, it remains unknown which anatomical site of stimulation [170] should be used for individual patients. Even if an optimal target for an individual patient were identified, ensuring accurate electrode placement, especially in areas with poor intrinsic MRI contrast, such as the thalamus, remains challenging [171–173]. Furthermore, contact localization remains a challenge, with many available tools but no consensus on assessing anatomic accuracy, especially in patients with preexisting structural brain injury causing distorted anatomy [174–177]. Similarly, for noninvasive brain stimulation techniques, the stimulation site should account for the individual patient’s underlying brain lesions and their associated network disconnections [178].

Questions persist regarding when to stimulate (e.g., how long after the brain injury, mornings and/or evenings, taking brain state fluctuation into account) and for how long (e.g., per session, per treatment period). Additionally, although noninvasive techniques, such as tACS, offer the opportunity to entrain neuronal oscillation to a specific frequency [122], which frequencies to target remains unknown. Furthermore, although much work has been done to model the current field to target a specific brain region on the basis of standardized atlases [175, 179], it is unknown whether such paradigms exert similar effects in the presence of extensive heterogeneous structural distortions commonly observed in the brains of patients with DoC [180]. It also remains unclear if concurrently administered medications hamper or facilitate brain stimulation efficacy. Finally, a key gap in the field of electromagnetic stimulation to promote recovery of consciousness is the lack of a large-sample randomized controlled trial.

### Proposal for Future Therapies

Generating individualized assessments of structural injury, functional network connectivity, and regional glucose metabolism may help inform the choice of a stimulation site. As our knowledge advances about how neural circuits within distributed brain networks encode and process information, strategies for targeted electromagnetic intervention may present themselves. Many stimulation systems now have sensing capabilities, which are needed to assess the effects of ongoing stimulation on neural activity. Separately, machine learning approaches may be useful for developing registration and segmentation pipelines that are robust to encephalomalacia and distortion and that precisely and reliably identify target structures (and electrode and lead location) in the brains of patients with DoC [173, 181, 182].

The development of neurophysiological biomarkers to measure electromagnetic treatment effects that occur independently of any behavioral change will help to guide future therapy. EEG properties (functional connectivity, spectral shifts) that are correlated with behavioral level of awareness may serve as candidate biomarkers by which electromagnetic therapies can be targeted and optimized [183]. Computational modeling of how stimulation paradigms applied to different sites affect underlying network physiology will be useful in designing treatment protocols with a higher chance of behavioral success [184].

Once treatment paradigms and methods of assessing behavioral or neurophysiologic end points are standardized, the variability in stimulation site can then be analyzed to optimize treatment effect. Such a strategy has already been applied successfully to rTMS treatment for depression [185]. It is also possible that combining bottom-up (e.g., VNS) and top-down (e.g., tES) therapies will provide synergistic effects with enhanced behavioral responses. Other future directions are to test simultaneous, multitarget stimulations and to use advanced brain imaging, such as diffusion MRI tractography and resting-state fMRI, to guide stimulation [15].

## Mechanical Therapies

### Transcranial Focused Ultrasound: State of the Science

The ability to focus low-intensity, subthreshold ultrasound toward subcortical targets allows ultrasound modulation to be conducted through an intact skull and scalp, permitting noninvasive stimulation [186, 187]. Low-intensity focused ultrasound pulsation (LIFUP) relies on direct mechanical effects on tissue rather than chemical or electromagnetic mechanisms. In preclinical studies, focused ultrasound has been used in rodents to ameliorate the effects of anesthesia and brain injury [188, 189]. A first-in-human study of LIFUP thalamic stimulation reported behavioral improvement in a single patient with acute posttraumatic DoC [190]. However, because the therapy was delivered only 19 days after injury, there is potential confounding by spontaneous recovery. A recent LIFUP study in three patients with chronic DoC provided further proof-of-principle evidence for its therapeutic potential, with two patients showing new behavioral responses after therapeutic stimulation [191]. Adverse events of LIFUP are still being investigated but potentially include the modulation of unintended targets and physical discomfort from the device during stimulation.

### Gaps in Knowledge

The use of focused ultrasound for patients with DoC is still in the early phases of development, and much remains unknown. Further research is needed on optimal anatomic targets, stimulation paradigms, the utility of adjuncts, such as microbubbles, and system design for robust chronic or intermittent clinical use.

### Proposal for Future Therapies

Future investigations with focused ultrasound should proceed down two pathways: one to optimize devices and protocols for the precise, durable modulation of neural tissue and the other to pinpoint appropriate modulation targets for patients with DoC. LIFUP research continues apace for myriad other uses, and its use in patients with DoC will undoubtedly benefit from (and hopefully contribute to) these advances.

## Sensory Therapies

### Tactile and Auditory Stimulation: State of the Science

Sensory stimulation therapies have been administered to patients with DoC for decades in rehabilitation settings [192]. They may be administered through any sensory modality, with tactile and auditory stimuli being the most common. The mechanistic rationale for this class of therapies is that environmental stimulation may enhance neural processing, support neuroplasticity, and thus promote reemergence of consciousness [193]. Sensory stimulation is postulated to reengage dormant subcortical networks that modulate arousal, resulting in reactivation of cortical networks that mediate awareness. Auditory stimulation is targeted toward activating auditory and language networks, as has been demonstrated in small placebo-controlled studies [194]. Music therapy aims to optimize the therapeutic impact of sensory stimulation by providing a live or recorded music stimulus [195], preferably performed in a personalized way by a music therapist [196–198], to activate neural networks that mediate attention, emotion, auditory processing, and self-awareness [199]. A recent meta-analysis suggested that music therapy may improve functional outcomes in patients with DoC [200].

Tactile and auditory therapies have an uncertain effect because they have thus far only been tested in small heterogeneous samples, along with variable therapeutic paradigms and outcome measures [193, 199, 201]. In the absence of compelling evidence from randomized controlled trials, the justification for these therapies rests on their safety and the reasonable assumption that sensory deprivation has deleterious effects on recovery.

### Vestibular Stimulation: State of the Science

There are three main methods of vestibular stimulation: motion devices (e.g., rotating chair), caloric vestibular stimulation (CVS), and galvanic vestibular stimulation (GVS). CVS consists of irrigating the external ear canal with warm or cold water. The subsequent change in afferent firing rate of the vestibular nerve simulates head and eye movement, which via brainstem and thalamic projections, produces responses in frontoparietal and striatal networks associated with arousal and goal-directed behavior [202]. GVS is a device that applies currents (0.1–3 mA) via two electrodes placed over the mastoid that provoke a change in equilibrium and nystagmus.

Previous studies investigated the effects of vestibular stimulation on various clinical conditions (e.g., sleep and mood disorders, schizophrenia, chronic pain), with positive results [203]. Other studies suggest that vestibular stimulation could serve as a sensory and cognitive enhancer [204, 205]. Different mechanisms have been suggested to explain its potential therapeutic effect, such as relocation of attention, multisensory integration, hemisphere-specific activation, and neurotransmitter release [203].

Only three studies have investigated the use of vestibular stimulation in patients with severe brain injuries. Two early studies demonstrated a correlation between electrooculographic recordings after CVS and the state of consciousness, but the duration of this effect was unclear [206, 207]. The third study showed time-locked behavioral improvements in two patients in a chronic MCS using a crossover design over 16–18 weeks of CVS and sham stimulation [208]. Vestibular stimulations are noninvasive, relatively inexpensive, and easy to implement. Mild side effects include motion sickness, vertigo, nausea, and vomiting.

### Gaps in Knowledge

The precise mechanisms underlying a potential therapeutic response to tactile, auditory, and vestibular therapies are unknown. Furthermore, knowledge about vestibular cortical representations is still limited, compared to other senses. Current evidence is based on case reports or small-scale studies, not yet replicated, and may be overestimating efficacy because of publication bias. Because most reported improvements were transient, whether sensory stimulation elicits sustained changes in the course of recovery is unknown.

### Proposal for Future Therapies

Well-controlled large-scale studies are needed, along with imaging or electrophysiologic recordings to confirm the preliminary results and elucidate the underlying mechanisms of tactile, auditory, and vestibular stimulation. Optimal protocols also need to be investigated, particularly with respect to the frequency and duration of sensory stimulation. Future studies should consider comparing efficacy of auditory therapies in which a patient actively participates (e.g., tapping a rhythm with one’s hand) with efficacy of auditory therapies in which a patient listens passively. Another future direction will be to determine whether auditory rhythms can induce brain rhythms—a neural entrainment similar to that observed with tACS [122]. New methods for CVS (e.g., wet air, near-infrared radiation) could be tested, and GVS could be used with virtual reality-based therapeutic interventions and rehabilitation.

## Regenerative Therapies

### Stem Cell, Neurogenesis, Gliogenesis, and Axonal Regrowth Therapies: State of the Science

Several therapeutic possibilities exist for using stem cells capable of neuronal differentiation in patients with DoC. These cells can be derived from adult neural stem cells, mesenchymal bone marrow stromal cells, umbilical cord blood, and induced pluripotent stem cells [209]. The application of this therapy to patients with DoC has been influenced by the development of platforms to test stem cell therapies in several other neurological diseases [210–212].

Few studies have evaluated the therapeutic effect of stem cells in patients with DoC. Two early-phase clinical trials in patients with traumatic DoC found that intravenous [213] or intrathecal [214] infusion of autologous bone marrow stromal cells was well tolerated at several different doses and possibly associated with faster rates of clinical improvement. Several additional case reports in children in a VS/UWS after anoxic injury showed clinical improvement following intravenous [215] or intracerebroventricular [216] infusion of umbilical cord blood. The safety profile of this therapy is not well established, and infusion-site reactions must be considered in addition to the potential of pluripotent cells to develop into malignancies [217].

### Gaps in Knowledge

Although efficient means of delivering neuronal precursor cells to brain tissue and evaluating their integration are being developed, the optimal approach for functional integration of stem cells into injured brain networks is unknown. Even in Parkinson's disease, with well-understood pathophysiology, discrete targets, and well-mapped circuitry, achieving functional integration of these cells has been difficult [210]. Furthermore, despite emerging insights into how the fate of stem cells is regulated [218, 219], the relative impact of stem cell therapies on neurogenesis, gliogenesis, and axonal regrowth has not been comprehensively characterized. The relative benefits of regenerative therapies that promote functional integration of neuronal precursor cells, as compared to those that provide trophic support for network plasticity, is also unknown.

### Proposal for Future Therapies

Given that patients with DoC frequently suffer widespread neuronal loss, the ability to deploy stem cells capable of reconstituting adult neurons is an appealing therapeutic option. Continuing to advance knowledge of the utility of regenerative therapies through rigorously testing and iteratively evaluating them will improve our chances of developing effective therapy for patients with DoC. Basic science progress, including the development of brain organoids that can be studied neurophysiologically [220], may offer more tractable models by which we can learn how to effectively use regenerative therapies. Cellular and molecular approaches to increasing the functional integration of stem cells induced to differentiate into neurons can be developed in vitro, optimized in animal models, and eventually tested in patients.

## Discussion and Future Directions

The development of effective consciousness-promoting therapies for patients with DoC will require a coordinated effort by the international community and a commitment to optimizing the design of clinical trials. We recommend that future studies implement multicenter, placebo-controlled, randomized, double-blind designs with complementary behavioral, neuroimaging, and electrophysiologic outcome measures to assess treatment efficacy. Mechanistic biomarkers that predict a therapeutic response are also needed to improve the efficiency of clinical trials by enrolling patients whose brain networks are amenable to therapeutic modulation. This precision medicine approach will require a broad range of methodological advances, including the rigorous characterization of patient endotypes [221].

Beyond advances in clinical trial design, we also recommend the development of new therapeutic approaches in which multiple therapies are administered concurrently to individual patients. Just as no single therapy is likely to be efficacious in all patients, it is possible that more than one therapeutic modality is needed to stimulate neural networks via synergistic mechanisms. For example, electromagnetic stimulation (e.g., rTMS) may be combined with pharmacologic stimulation [222], or electromagnetic top-down stimulation (e.g., tES) with bottom-up approaches (e.g., transauricular VNS), administered either concurrently or consecutively. We encourage the development of adaptive clinical trial designs featuring conditional therapeutic additions or changes based on the patient’s clinical evolution.

For these new approaches to reach their full potential, we will need a unifying conceptual framework—one that accounts for the diverse pathophysiologic mechanisms underlying DoC. This conceptual framework will help guide the development of surrogate end points, or pharmacodynamic biomarkers, of therapeutic efficacy in early-stage clinical trials (i.e., phases 1 and 2). When testing whether a new therapy is engaging its target, it is likely that subclinical responses will be detectable before behavioral responses [17, 141–143, 223]. Pharmacodynamic biomarkers derived from EEG [17, 183, 223, 224], fMRI [17, 225], positron emission tomography [225], TMS–EEG [141–143], or near-infrared spectroscopy [226] can thus be used to measure brain responses to new therapies, identify optimal dosing regimens, and inform the design of phase 3 trials that aim to detect behavioral and functional responses.

In parallel with the need for surrogate measures in early-phase trials, there are fundamental unanswered questions about the optimal outcome measures to use for phase 3 trials that enroll patients with DoC. Historically, the Glasgow Outcome Scale-Extended (GOSE) [227] has been the outcome measure recommended by regulatory agencies for phase 3 clinical trials of patients with severe brain injuries [228, 229]. However, the GOSE is an ordinal eight-point scale with outcome categories that do not provide the granular assessment of consciousness or cognitive function that may be required to detect subtle, yet clinically meaningful, therapeutic effects. A patient who transitions from a VS/UWS to a low-level MCS, for example, would not be defined as a treatment responder by using the GOSE because the score would remain a 2 [230]. Indeed, the reliance on the GOSE as an outcome measure has been proposed as a contributing factor to the high failure rate of phase 3 clinical trials in patients with severe brain injuries [229]. The Disability Rating Scale [231] provides a more comprehensive assessment of functional outcome and was used in the phase 3 trial of amantadine [6], but the Disability Rating Scale does not account for behavioral changes in the visual and auditory domains that would be captured by the Coma Recovery Scale-Revised [23]. Yet even if future phase 3 trials include additional behavioral and cognitive outcome measures derived from the Coma Recovery Scale-Revised and the Confusion Assessment Protocol [232], fundamental questions remain, such as the following: (1) What is the minimal clinically important difference [233] for outcome measures that assess patients with DoC? (2) Does the minimal clinically important difference depend on the level of consciousness at the time of trial enrollment? and (3) Are outcome measures that rely on overt behaviors suboptimal for patients with covert consciousness, who may only be able to communicate via brain–computer interfaces [33]? Answering these questions may require new partnerships between clinicians, investigators, ethicists, recovered patients, caregivers, and regulatory agencies.

Another key consideration in future clinical trial design will be the timing of enrollment, particularly for patients with cognitive motor dissociation [30] (i.e., active command-following on task-based fMRI or EEG) or covert cortical processing [10] (i.e., passive responses to language or music on stimulus-based fMRI or EEG). Emerging evidence suggests that these two groups of patients have a better chance of long-term functional recovery than do patients without responses on task-based or stimulus-based diagnostic tests [26, 234], which may also suggest an increased receptivity to therapeutic stimulation. Investigators will have to consider whether these patients should be analyzed as prespecified subgroups in future studies and whether a transition from unresponsiveness to cognitive motor dissociation or covert cortical processing should be defined as a favorable therapeutic response.

In summary, the future development of all five classes of therapeutic modalities investigated in this gap analysis will require multicenter trials to achieve adequate statistical power to test hypotheses about therapeutic efficacy. We call for the creation of a global DoC clinical trials network to support this long-term goal. Central to this international effort will be the selective enrollment of patients based on their physiological receptivity to targeted therapies [17, 18, 94, 95], as well as the implementation of new pharmacodynamic biomarkers and standardized outcome measures for the comprehensive evaluation of brain function, behavior, and cognition. Advances in clinical trial design and precision medicine are essential for the future development of therapies that will improve the lives of patients with DoC.

## Supplementary Information

Below is the link to the electronic supplementary material.

Supplementary file1 (DOCX 20 kb)

Supplementary file1 (XLSX 13 kb)
