# Supplementary material for: Therapies to Restore Consciousness in Patients with Severe Brain Injuries: A Gap Analysis and Future Directions
Source: Neurocrit Care. 2021 Jul 8;35(Suppl 1):68–85. doi: 10.1007/s12028-021-01227-y (PMC8266715; doi:10.1007/s12028-021-01227-y)
Supplement: Supplementary file 1 — Supplementary file1 (DOCX 20 kb) [file 12028_2021_1227_MOESM1_ESM.docx]

Supplementary Table 1. Acknowledgement of Curing Coma Campaign collaborators participating in the overall program

| Venkatesh | Aiyagari |
| --- | --- |
| Yama | Akbari |
| Fawaz | Al-Mufti |
| Sheila | Alexander |
| Anne | Alexandrov |
| Ayham | Alkhachroum |
| Mary Kay | Bader |
| Ram | Balu |
| Megan | Barra |
| Rachel | Beekman |
| Kathleen | Bell |
| Erta | Beqiri |
| Thomas | Bleck |
| Yelena | Bodien |
| Varina | Boerwinkle |
| Melanie | Boly |
| Alexandra | Bonnel |
| Emery | Brown |
| Eder | Caceres |
| Josh | Cain |
| Elizabeth | Carroll |
| Emilio G. | Cediel |
| Sherry | Chou |
| Giuseppe | Citerio |
| Jan | Claassen |
| Angela | Comanducci |
| Chad | Condie |
| Katie | Cosmas |
| Claire | Creutzfeldt |
| Neha | Dangayach |
| Michael | DeGeorgia |
| Caroline | Der-Nigoghossian |
| Masoom | Desai |
| Michael | Diringer |
| Brian | Edlow |
| Satoshi | Egawa |
| Ari | Ercole |
| Anna | Estraneo |
| Salia | Farrokh |
| Davinia | Fernandez-Espejo |
| Joseph | Fins |
| Brandon | Foreman |
| Jennifer | Frontera |
| Joe | Giacino |
| Christie | Gibbons |
| Emily | Gilmore |
| Ursula | Gorska |
| Olivia | Gosseries |
| Theresa | Green |
| David | Greer |
| Mary | Guanci |
| Cecil | Hahn |
| Ryan | Hakimi |
| Flora | Hammond |
| Daniel | Hanley |
| Jed | Hartings |
| Ahmed | Hassan |
| Raimund | Helbok |
| Claude | Hemphill |
| Holly | Hinson |
| Karen | Hirsch |
| Sarah | Hocker |
| Xiao | Hu |
| Peter | Hu |
| Andrew | Hudson |
| Theresa | Human |
| David | Hwang |
| Luke | James |
| Anna | Janas |
| Morgan | Jones |
| Sheryl | Katta-Charles |
| Emanuela | Keller |
| Maggie | Keogh |
| Jenn | Kim |
| Keri | Kim |
| Hannah | Kirsch |
| Nerissa | Ko |
| Daniel | Kondziella |
| Walter | Koroshetz |
| Natalie | Kreitzer |
| Julie | Kromm |
| Abhay | Kumar |
| Pedro | Kurtz |
| Steven | Laureys |
| Ariane | Lewis |
| John | Liang |
| Geoff | Ling |
| Sarah | Livesay |
| Andrea | Luppi |
| Lori | Madden |
| Craig | Maddux |
| Dea | Mahanes |
| Shraddha | Mainali |
| Marcello | Massimini |
| Jaffa | Matthew |
| Stephan | Mayer |
| Victoria | McCredie |
| Molly | McNett |
| David | Menon |
| Geert | Meyfroidt |
| Dick | Moberg |
| Asma | Moheet |
| Martin | Monti |
| Chris | Morrison |
| Susanne | Muehlschlegel |
| Lionel | Naccache |
| Masao | Nagayama |
| Girija | Natarajan |
| Virginia | Newcombe |
| Paul | Nyquist |
| DaiWai | Olson |
| Marwan | Othman |
| Adrian | Owen |
| Soojin | Park |
| Melissa | Pergakis |
| Len | Polizzotto |
| Nader | Pouratian |
| Marilyn | Price Spivack |
| Javier | Provencio |
| Louis | Puybasset |
| Chethan | Rao |
| Verena | Rass |
| Chiara | Robba |
| Courtney | Robertson |
| Benjamin | Rohaut |
| John | Rolston |
| Mario | Rosanova |
| Eric | Rosenthal |
| Mary Beth | Russell |
| Gisele | Sampaio |
| Leandro | Sanz |
| Simone | Sarasso |
| Aarti | Sarwal |
| Nicolas | Schiff |
| David | Seder |
| Vishank Arun | Shah |
| Angela | Shapshak |
| Tarek | Sharshar |
| Lori | Shutter |
| Jacobo | Sitt |
| Beth | Slomine |
| Peter | Smielewski |
| Wade | Smith |
| Sam | Snider |
| Lennart | Spindler |
| Emmanuel | Stamatakis |
| Alexis | Steinberg |
| Robert | Stevens |
| Jose | Suarez |
| Shaurya | Taran |
| Aurore | Thibaut |
| Zachary | Threlkeld |
| Daniel | Toker |
| Stephen | Trevick |
| Alexis | Turgeon |
| Panos | Varelas |
| Paul | Vespa |
| Walter | Videtta |
| Henning | Voss |
| Amy | Wagner |
| John | Whyte |
| Briana | Witherspoon |
| Aleksandra (Sasha) | Yakhkind |
| Michael | Young |
| Ross | Zafonte |
| Darin | Zahuranec |
| Chris | Zammit |
| Wendy | Ziai |
| Lara | Zimmerman |
| Elizabeth | Zink |
